# ﻿Unveiling two new species of *Trichoderma* (Hypocreales, Hypocreaceae) that cause green mold disease on *Strophariarugosoannulata* from Guizhou Province, China

**DOI:** 10.3897/mycokeys.110.134154

**Published:** 2024-11-22

**Authors:** Entaj Tarafder, Zhang Wenjun, Samantha C. Karunarathna, Abdallah M. Elgorban, Man Huilian, Wu Nan, Xiangyu Zeng, Wang Yong, Feng-Hua Tian

**Affiliations:** 1 Department of Plant Pathology, College of Agriculture, Guizhou University, Guiyang, China Tarafder Guiyang China; 2 Institute of Edible Mushroom, Guizhou University, Guiyang, China Jilin Agricultural University Changchun China; 3 Engineering Research Center of Chinese Ministry of Education for Edible and Medicinal Fungi, Jilin Agricultural University, Changchun 130118, China Guizhou University Guiyang China; 4 Center for Yunnan Plateau Biological Resources Protection and Utilization, College of Biological Resource and Food Engineering, Qujing Normal University, Qujing, Yunnan 655011, China Qujing Normal University Qujing China; 5 Department of Botany and Microbiology, College of Science, King Saud University, Riyadh 11451, Saudi Arabia King Saud University Riyadh Saudi Arabia; 6 School of Life Science and Technology, Heilongjiang Bayi Agricultural Reclamation University, Daqing 163316, China Heilongjiang Bayi Agricultural Reclamation University Daqing China

**Keywords:** Ascomycetes, novel taxa, pathogen, phylogeny, taxonomy

## Abstract

*Strophariarugosoannulata* is an important edible mushroom in China, but green mold disease has caused significant production and economic losses. In this study, two new pathogens *Trichodermastrophariensis* and *T.viridistromatis* were identified as the causal agents of this disease. During October-November 2023, six strains of the fungal pathogen were isolated from infected fruiting bodies of *S.rugosoannulata* and identified based on morphological characteristics and molecular phylogenetic analyses of internal transcribed spacer (nrITS), the second largest RNA polymerase II subunit (*rpb2*) and the partial translation elongation factor 1-alpha (*tef1-α*) region. The representative isolates of the pathogenic green mold *Trichoderma* species were used to perform a pathogenicity test with spore suspensions, resulting in symptoms similar to those observed in the cultivated field. The same pathogens were successfully re-isolated, thereby fulfilling Koch’s postulates. Detailed morphological descriptions, illustrations, culture characteristics, and comparisons with morphologically similar and closely related species are provided.

## ﻿Introduction

*Strophariarugosoannulata* (Wine-cap mushroom), a renowned edible mushroom, also known as Daqiugaigu in Chinese, has been widely cultivated in Poland, Germany, Russia and the United States (Huang et al. 2023). China imported a strain of *S.rugosoannulata* from Poland in the 1980s and began widespread cultivation in the 1990s (Yan et al. 2020). In recent years, *S.rugosoannulata* has been rapidly promoted and widely cultivated throughout China (Gu et al. 2024). With the increasing scale of cultivation, the annual yield of *S.rugosoannulata* in China has exceeded 210,000 tons per year (Huang et al. 2023). However, the emergence of various diseases during the cultivation of *S.rugosoannulata* has driven researchers to intensify their efforts to optimize its growth conditions. Our investigation observed green mold disease on the soil surface and fruiting bodies of *S.rugosoannulata* from three different localities. This disease incidence can lead to mushroom rot and a decline in yield and quality. The dedication of researchers to addressing this issue is a reassuring sign for the future of *S.rugosoannulata* cultivation.

Green mold disease is a major prevalent disease that frequently arises during mushroom development and is characterized by green, villiform mycelia on the surface (Li et al. 2013). *Trichoderma* Pers. (Hypocreales, Ascomycota) is a saprobic fungus found in soil, healthy plants, wood, and other fungi and plays a crucial role as the causative agent of green mold disease. *Trichoderma* species are widely used to combat fungal pathogens (Hasan et al. 2012; Liu et al. 2012; Li et al. 2013; Abo-Elyousr et al. 2014; Poveda et al. 2019), produce antibiotics, enzymes, and biofuel (Degenkolb et al. 2008; Jun et al. 2011; Wijayawardene et al. 2022). Additionally, *Trichoderma* species contribute to the bioremediation of xenobiotic compounds in water and soil (Katayama and Matsumura 1993; Harman et al. 2004; Ezzi and Lynch 2005). Currently, *Trichoderma* comprises more than 500 species globally, based on the literature search (Jaklitsch 2009; Jaklitsch and Voglmayr 2015; Jambhulkar et al. 2024), legitimate names in the Mycobank (https://www.mycobank.org.) and in the Species Fungorum database (www.speciesfungorum.org; accessed on 23 October 2024).

*Trichoderma* has two types of species with differing ascospore colours, namely hyaline and green ascospores. Chaverri and Samuels (2004) pioneered comprehensive research on green-spored *Trichoderma* species, providing foundational insight into their taxonomy and systematics. Subsequently, Jaklitsch and Voglmayr (2015) proposed a comprehensive classification primarily based on molecular phylogenetic analyses rather than the color of ascospores, dividing them into six subclades: *Ceramicum*, *Chlorosporum*, Harzianum, *Helium*, *Spinulosum*, and *Strictipile*. However, other researchers have not recognized this classification, largely due to the inconsistencies between molecular sequence data and morphological characteristics, as highlighted by Chen and Zhuang (2017). Bustamante et al. (2021) used multi-locus phylogenetic analyses alongside four DNA-based approaches to accurately delimit species within the *Trichoderma* Harzianum lineage, including most green-spored species.

The present study was conducted based on the pathogen of the green mold disease, aiming to characterize and identify the isolates. Six isolates were isolated from soil samples and fruiting bodies of *S.rugosoannulata* cultivated fields in three different regions of Guizhou Province, China. The study described two new species and compared their morphological characteristics among closely related species. A combined dataset of ITS, *rpb2*, and *tef1-α* was used for the thorough phylogenetic analyses, ensuring the reliability of the results.

## ﻿Materials and methods

### ﻿Pathogen collection, isolation, and maintenance

Infected fruiting bodies of *S.rugosoannulata* were collected from mushroom-cultivated fields at Baiyun and Shuicheng counties (23°4'23.6352"N, 120°37'39.7812"E and 24°55'39.936"N, 121°11'30.264"E), Guizhou Province, China in October-November 2023. Field photographs of the fresh specimens were taken with a Canon EOS 1200D (Canon, Japan) or Sony DSC-W830 (Sony, Japan) camera. The specimens were packed in aluminium foil and transferred to the Plant Pathology Laboratory at Guizhou University for isolation. Fungal pathogens on infected fruiting bodies were isolated using the spread plate and tissue isolation method following Wang et al. (2019). Purified cultures were incubated on potato dextrose agar (PDA), malt extract agar (MEA), and synthetic low nutrient agar (SNA) plates at 25, 30, and 35 °C. The holotype specimen was deposited in the Herbarium of the Department of Plant Pathology, Agricultural College, Guizhou University (HGUP). All single ex-type strains were deposited in the Culture Collection of the Guizhou University, China (GUCC) Department of Plant Pathology at Agriculture College and maintained in 25% (v/v) glycerol at –80 °C for long-term preservation (Zeng et al. 2022). Index Fungorum numbers were registered for the new taxa (https://www.indexfungorum.org/names/Names.asp).

### ﻿Pathogenicity assays

A pathogenicity test was conducted by inoculating fungal mycelial blocks and spore suspensions from six strains isolated from Baiyun, Shuicheng, and Anshun counties onto the soil surface and fruiting bodies of *S.rugosoannulata*, following the updated protocol of Tian et al. (2017). All strains were incubated at 25 °C for 10 days. Control checks (CK) included PDA blocks and distilled water, replacing the mycelial blocks and spore suspensions. Photographs of the inoculated soils were taken after one, seven, and 10 days to monitor the development of any green mycelia. After the 10-day incubation, fungal pathogens were re-examined and re-isolated from the diseased areas to fulfill Koch’s postulates, ensuring accurate identification of pathogenicity (Zhang et al. 2015; Xie et al. 2024). The experiment was repeated three times to validate the results and account for variability.

### ﻿Morphological studies

Micro-morphological observations were performed from culture photo­graphs of fresh stromata, which were taken using an ultra-depth field stereomicroscope (digital microscope system Keyence VHX-7000) to illustrate the macrostructures. Sections were made using a stereomicroscope (Leica DM2500) and mounted in water or a rehydrated 5% KOH solution. The cultures were incubated at 25 °C in darkness (Põldmaa 2011; Wei et al. 2024). Approximately 30 morphological measurements of new species were made for each feature using the ZEN 3.0 (blue edition) (Jena, Germany) software (Zeiss Scope 5 with color camera AxioCam 208) with differential interference contrast (DIC) optics to observe the morphological characteristics (Jaklitsch and Voglmayr 2015; Fu et al. 2024; Zeng et al. 2024). Colony characteristics, i.e., color and texture on PDA (Potato dextrose agar; 200 g potatoes, 20 g dextrose, 20 g agar per L), MEA (malt extract agar; 30 g malt extract, 5 g mycological peptone, 15 g agar per L) and synthetic low nutrient agar (SNA) plates at 25, 30 and 35 °C were observed and noted over 14 days.

### ﻿Molecular studies

#### ﻿DNA extraction, Polymerase Chain Reaction (PCR) and sequencing

The genomic DNA was extracted from the colony of the isolates cultured at 25 °C, PDA for seven days using a CwBiotech Plant Genomic DNA Kit (Changping, Beijing, China) following the manufacturer’s protocol.

The internal transcribed spacer (nrITS), the second largest RNA polymerase II subunit (*rpb2*) and the partial translation elongation factor 1-alpha (*tef1-α*) regions were amplified using the primer pairs ITS5/ITS4, EF1-728F/TEF1LLErev, and fRPB2-5F/fRPB2-7cR, respectively (White et al. 1990; Carbone and Kohn 1999; Liu et al. 1999; Jaklitsch et al. 2005). A 25 mL reaction mixture containing 1.6 mL dNTP mix (2.5 mM/mL), 0.2 mL Taq polymerase (5 U/mL), 2 mL polymerase buffer (10 /mL), 1 ml forward and reverse primers (10 mM/mL), and 1 mL DNA template was used for PCR experiments. Amplifications were carried out in a T100^TM^ Thermal Cycler (BIO-RAD), which was configured for an initial denaturation at 95 °C for 3 minutes, followed by 34 cycles of 1 minute at 95 °C, 30 seconds at 55 °C, 1-minute extension at 72 °C, and a final extension at 72 °C for 10 minutes. Sangon Biotech (Shanghai) Co., Ltd. sequenced PCR products using the same PCR primers used in amplification operations. The newly generated sequences were checked with BioEdit v.7.2.5 (Hall 1999) and deposited in the NCBI GenBank nucleotide database for future reference.

The amplified sequences were subjected to BLASTn searches in the GenBank nucleotide database for comparison. Subsequently, closely related sequences of the taxa exhibiting zero E-values were retrieved from the database to generate the dataset. Besides, the sequences used by earlier studies on *Trichoderma* (Zeng et al. 2022) were also obtained from the database to prepare the final dataset (Table 1).

### ﻿Dataset representation

Sequences of the closely related taxa with zero E-value were searched from the BLASTn analyses in the NCBI GenBank nucleotide database. A preliminary BLAST search with the newly amplified sequences of the collected specimens showed the highest sequence similarity with the members of the *Trichoderma* Pers. Hence, a dataset was prepared based on the highest-scored hits of the BLAST search plus the datasets used in the earlier studies on *Trichoderma* (Zeng et al. 2022).

### ﻿Sequence alignment and phylogenetic analyses

The newly generated reverse and forward sequences were reassembled manually using BioEdit version 7.0.5.3 (Hall 1999) and were aligned with MAFFT v.7.427 (Katoh et al. 2019) in an online platform (https://www.ebi.ac.uk/Tools/msa/mafft/). The aligned sequences were imported to MEGA v.7.0 (Kumar et al. 2016) for manual improvement and trimming of both ends.

A quick phylogenetic analyses of DNA fragments (ITS, *rpb2* and *tef1-α*) from 128 strains were performed with alignments and associated data matrices, including six isolates in this study (GUCC TB1117, GUCC TB1118, GUCC TB1119, GUCC TB1120, GUCC TB1121 and GUCC TB1122) and 122 reference strains (Table 1) by using offline software ‘One-click Fungal Phylogenetic Tool’ (OFPT-https://ofpt.guhongxin.com) following its default protocol (Zeng et al. 2023). The final Maximum likelihood analysis was performed with RAxML-HPC2 v. 8.2.12 (Stamatakis 2014) on the CIPRES Science Gateway platform using the GTR+I+G model with 1,000 bootstrap replicates and Bayesian analyses were conducted with MrBayes v.3.2.2 (Ronquist et al. 2012) using MCMC methods (Geyer 1991) under a GTR+I+G model. Markov chains were run for 2 × 106 generations, saving a tree every 100^th^ generation with all the remaining parameters set to default. Bayesian analyses reached a standard deviation of split frequency of 0.0048 at the end of the specified number of generations. For both analyses, the initial 25% of trees recovered (10,000 trees) were excluded as the burn-in, while the remaining 30,002 trees were utilized to estimate the posterior probabilities for the group. ML bootstrap values (MLBS) ≥ 70% and Bayesian posterior probabilities (PP) values ≥ 0.95 are displayed in the phylogenetic tree. The resulting trees were visualized in FigTree v1.4.3 (Rambaut 2016).

**Table 1.** Names, strain numbers, locations, and corresponding GenBank accession numbers of the taxa used in the phylogenetic analysis.

Species	Strain	Geographic origin	GenBank Accession Numbers		
			ITS	*rpb2*	*tef1-α*
*T.achlamydosporum*	YMF 1.06226	China	MN977791	MT052180	MT070156
*T.aerugineum*	CBS 120541 T	Austria	FJ860720	FJ860516	FJ860608
*T.afarasin*	DIS 314F	Cameroon	FJ442259	FJ442778	FJ463400
*T.afroharzianum*	CBS 466.94	Netherlands	KP009262	KP009150	KP008851
*T.aggregatum*	HMAS 248863	China	KY687946	KY688001	KY688062
*T.aggressivum*	CBS 100525	United Kingdom	-	AF545541	AF534614
*T.alni*	CBS 120633 T	United Kingdom	EU518651	EU498349	EU498312
*T.alpinum*	HMAS 248821 T	China	KY687906	KY687958	KY688012
*T.amazonicum*	IB 95	Peru	-	HM142368	HM142377
*T.anaharzianum*	YMF 1.00383	China	MH113931	MH158995	MH183182
*T.asiaticum*	YMF 1.00352	China	MH113930	MH158994	MH183183
*T.atrobrunneum*	S3	Italy	-	KJ665241	KJ665376
*T.atrogelatinosum*	CBS 237.63 T	New Zealand	MH858272	KJ842201	KJ871083
*T.attinorum*	LESF 236	USA	-	KT278971	KT279039
*T.aureoviride*	CPK 2848	Germany	FJ860733	FJ860523	FJ860615
*T.azevedoi*	CEN1422 T	Brazil	MK714902	MK696821	MK696660
*T.bannaense*	HMAS 248840 T	China	KY687923	KY687979	KY688037
*T.breve*	HMAS 248844 T	China	KY687927	KY687983	KY688045
*T.brevicrassum*	HMAS 248871 T	China	KY687954	KY688008	KY688064
*T.britannicum*	CBS 253.62 T	United Kingdom	MH858149	KF134787	KF134796
*T.brunneoviride*	CBS 121130	Germany	EU518659	EU498357	EU498316
*T.byssinum*	HMAS 248838 T	China	KY687921	KY687977	KY688035
*T.catoptron*	GJS 02-76 T	Sri Lanka	AY737766	AY391900	AY391963
*T.ceraceum*	GJS 95-159 T	North Carolina	AF275332	AF545508	AF534603
*T.ceramicum*	CBS 114576 T	Austria	FJ860743	FJ860531	FJ860628
*T.ceratophylli*	YMF 1.04621	China	MK327581	MK327580	MK327579
*T.cerinum*	S357	France	-	KF134788	KF134797
*T.chlamydosporicum*	HMAS 248850	China	KY687933	KY687989	KY688052
*T.chlorosporum*	GJS 88-33 T	USA	-	AY391903	AY391966
*T.christiani*	CBS 132572 T	Spain	-	KJ665244	KJ665439
*T.chromospermum*	HMAS 252535	China	KF923304	KF923315	KF923292
*T.cinnamomeum*	GJS 97-237	USA	AY737759	AY391920	AY391979
*T.compactum*	CBS 121218 T	China	-	KF134789	KF134798
*T.concentricum*	HMAS 248833 T	China	KY687915	KY687971	KY688027
*T.corneum*	GJS 97-82 ET	Thailand	-	KJ665252	KJ665455
*T.costaricense*	PC 21 T	Costa Rica	AY737754	AY391921	AY391980
*T.cremeoides*	S112 T	Italy	-	KJ665253	KJ665456
*T.cremeum*	GJS 91-125 T	USA	AY737760	AF545511	AF534598
*T.cuneisporum*	GJS 91-93 T	USA	AY737763	AF545512	AF534600
*T.dacrymycellum*	WU 29044	Germany	FJ860749	FJ860533	FJ860633
*T.danicum*	CBS 121273 T	Denmark	FJ860750	FJ860534	FJ860634
*T.epimyces*	CBS 120534 T	Austria	EU518663	EU498360	EU498320
*T.estonicum*	GJS 96-129 T	Estonia	AY737767	AF545514	AF534604
*T.ganodermatis*	HMAS 248856	China	KY687939	KY687995	KY688060
*T.gelatinosum*	GJS 88-17	France	AY737775	AF545516	AF534579
*T.gliocladium*	CBS 130009 T	Italy	MH865622	KJ665271	KJ665502
*T.guizhouense*	S278	Croatia	-	KF134791	KF134799
*T.hainanense*	HMAS 248837 T	China	KY687920	KY687976	KY688033
*T.harzianum*	CBS 226.95 T	Austria	AY605713	AF545549	AF534621
*T.hausknechtii*	CBS 133493 T	France	-	KJ665276	KJ665515
*T.helicolixii*	CBS 133499 T	Greece	-	KJ665278	KJ665517
*T.helicum*	DAOM 230021	Austria	-	DQ087239	KJ871125
*T.hirsutum*	HMAS 248834 T	China	KY687916	KY687972	KY688029
*T.hunanense*	HMAS 248841 T	China	KY687924	KY687980	KY688039
*T.hymenopellicola*	GUCC202008	China	MZ330754	ON088663	ON102007
*T.hymenopellicola*	GUCC202009	China	MZ330755	ON088664	ON102008
*T.hymenopellicola*	GUCC202010	China	MZ330756	ON088661	ON102005
*T.hymenopellicola*	GUCCTB626	China	ON074580	ON088662	ON102006
*T.hymenopellicola*	GUCCTB625	China	ON074583	-	ON102011
*T.inaequilaterale*	YMF 1.06203	China	MN977795	MT052186	MT070152
*T.ingratum*	HMAS 248822 T	China	KY687917	KY687973	KY688018
*T.inhamatum*	CBS 273.78 T	Colombia	-	FJ442725	AF348099
*T.italicum*	CBS 132567 T	Italy	-	KJ665282	KJ665525
*T.jaklitschii*	CP61-2 T	Peru	-	MW480149	MW480140
*T.lentiforme*	DIS 94D	Peru	-	FJ442749	FJ463379
*T.lentinulae*	CGMCC 3.19847 T	China	-	MN605867	MN605878
*T.liberatum*	HMAS 248831 T	China	KY687913	KY687969	KY688025
*T.linzhiense*	HMAS 248846 T	China	KY687929	KY687985	KY688047
*T.lixii*	CBS 110080 T	USA	AF443920	KJ665290	FJ716622
*T.longibrachiatum*	CBS 816.68 T	Austria	Z31019	DQ087242	EU401591
*T.longifialidicum*	LESF 552	USA	-	KT278955	KT279020
*T.longipile*	DAOM 177227 T	Austria	AY865630	AF545550	AF534622
*T.longisporum*	HMAS 248843	China	KY687926	KY687982	KY688043
*T.lycogaloides*	WU 32096 T	French Guiana	-	KF134792	KF134800
*T.parepimyces*	CBS 122769 T	Austria	FJ860800	FJ860562	FJ860664
*T.parestonicum*	CBS 120636 T	Austria	FJ860803	FJ860565	FJ860667
*T.peberdyi*	CEN1426 T	Brazil	MK714906	MK696825	MK696664
*T.peruvianum*	CP15-2 T	Peru	-	MW480153	MW480145
*T.perviride*	HMAS 273786 T	China	-	KX026962	KX026954
*T.phyllostachydis*	CBS 114071 T	Austria	FJ860809	FJ860570	FJ860673
*T.pinicola*	SFC20130926-S233 T	South Korea	MH050354	MH025993	MH025981
*T.pleuroti*	CBS 124387 T	USA	-	HM142372	HM142382
*T.pleuroticola*	CBS 124383 T	USA	-	HM142371	HM142381
*T.polypori*	HMAS 248855 T	China	KY687938	KY687994	KY688058
*T.priscilae*	CBS 131487 T	Austria	-	KJ665333	KJ665691
*T.propepolypori*	YMF 1.06224	China	MN977789	MT052181	MT070158
*T.pseudoasiaticum*	YMF 1.06200 T	China	MN977792	MT052183	MT070155
*T.pseudocandidum*	PC 59 T	Costa Rica	AY737757	AY391899	AY737742
*T.pseudodensum*	HMAS 248828 T	China	KY687910	KY687967	KY688023
*T.pseudogelatinosum*	CNU N309 T	South Korea	-	HM920173	HM920202
*T.purpureum*	HMAS 273787 T	China	-	KX026961	KX026953
*T.pyramidale*	CBS 135574 T	Italy	-	KJ665334	KJ665699
*T.rifaii*	DIS 337F ET	Panama	-	FJ442720	FJ463321
*T.rosulatum*	HMAS 252548	China	KF729995	KF730005	KF729984
*T.rufobrunneum*	HMAS 266614 T	China	KF729998	KF730010	KF729989
*T.rugulosum*	SFC20180301-1 T	South Korea	MH050353	MH025986	MH025984
*T.shennongjianum*	HMAS 245009	China	-	KT735259	KT735253
*T.silvae-virgineae*	CBS 120922	Austria	-	FJ860587	FJ860696
*T.simile*	YMF 1.06201	China	MN977793	MT052184	MT070154
*T.simmonsii*	S7	Italy	-	KJ665337	KJ665719
*T.simplex*	HMAS 248842 T	China	KY687925	KY687981	KY688041
*T.sinuosum*	CPK 1595	Austria	FJ860838	FJ179619	FJ860697
*T.solum*	HMAS 248848 T	China	KY687931	KY687987	KY688050
*T.spinulosum*	CBS 311.50 T	Austria	FJ860844	FJ860591	FJ860701
*T.spirale*	DAOM 183974 T	Thailand	EU280068	AF545553	EU280049
*T.stipitatum*	HMAS 266612	China	KF730002	KF730011	KF729990
*T.stramineum*	GJS 02-84 T	Sri Lanka	AY737765	AY391945	AY391999
*T.strictipile*	CPK 1601	Austria	-	FJ860594	FJ860704
***T.strophariensis***	**GUCC TB1117 T**	**China**	**PP920011**	**PP954941**	**PP954947**
***T.strophariensis***	**GUCC TB1118**	**China**	**PP920012**	**PP954942**	**PP954948**
***T.strophariensis***	**GUCC TB1119**	**China**	**PP920013**	**PP954943**	**PP954949**
*T.subazureum*	YMF 1.06207	China	MN977799	MT052190	MT070148
*T.subuliforme*	YMF 1.06204	China	MN977796	MT052187	MT070151
*T.sulawesense*	GJS 85-228	USA	-	AY391954	AY392002
*T.surrotundum*	GJS 88-73 T	USA	AY737769	AF545540	AF534594
*T.tawa*	GJS 97-174 T	Thailand	AY737756	AY391956	AY392004
*T.tenue*	HMAS 273785 T	China	-	KX026960	KX026952
*T.thailandicum*	GJS 97-61 T	Thailand	AY737772	AY391957	AY392005
*T.thelephoricola*	CBS 120925	Austria	FJ860858	FJ860600	FJ860711
*T.tibetense*	HMAS 245010	China	-	KT735261	KT735254
*T.tomentosum*	CBS 120637	Austria	-	FJ860532	FJ860629
*T.tropicosinense*	HMAS 252546	China	KF923302	KF923313	KF923286
*T.undatipile*	HMAS 248854	China	KY687937	KY687993	KY688056
*T.velutinum*	CPK 298 T	Nepal	-	KF134794	KJ665769
*T.vermifimicola*	CGMCC 3.19694 T	China	MN594473	MN605871	MN605882
*T.virens*	DAOM 167652 T	USA	EU330955	AF545547	AF534619
*T.virescentiflavum*	PC 278	Costa Rica	AY737768	AY391959	AY392007
***T.viridistromatis***	**GUCC TB1120 T**	**China**	**PP922277**	**PP954944**	**PP954950**
***T.viridistromatis***	**GUCC TB1121**	**China**	**PP926290**	**PP954945**	**PP954951**
***T.viridistromatis***	**GUCC TB1122**	**China**	**PP922285**	**PP954946**	**PP954952**
*T.xixiacum*	CGMCC 3.19697 T	China	MN594476	MN605874	MN605885
*T.zayuense*	HMAS 248835 T	China	KY687918	KY687974	KY688031
*T.zelobreve*	CGMCC 3.19695 T	China	MN594474	MN605872	MN605883
*T.zeloharzianum*	YMF 1.00268	China	MH113932	MH158996	MH183181

Note: Newly sequenced strains are shown in bold. T denotes type cultures.

## ﻿Results

### ﻿Pathogenicity tests

**Figure 1.**
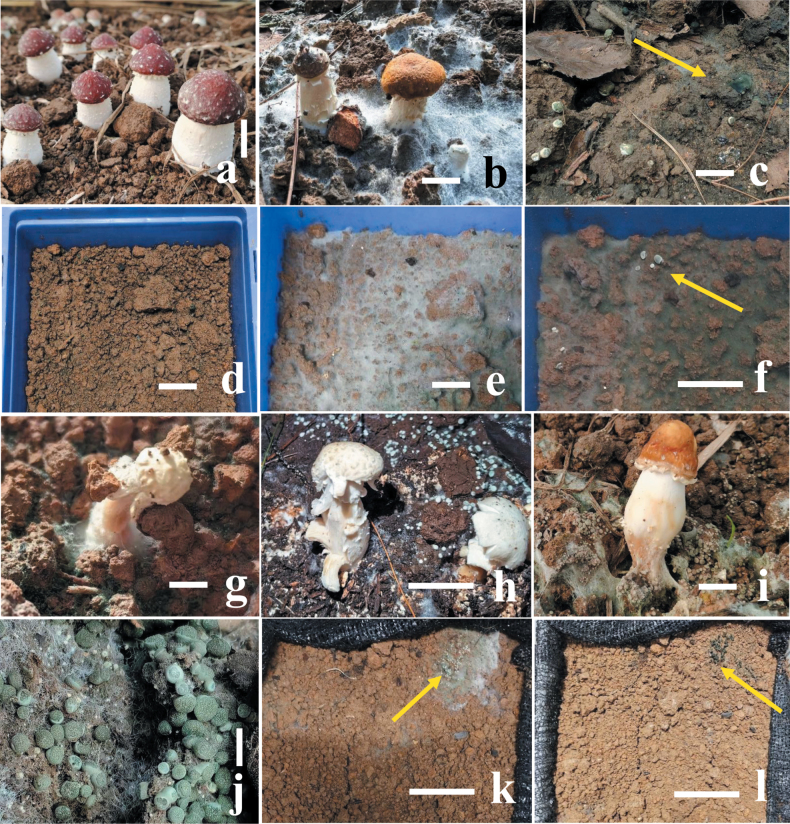
Field symptoms of green mold disease on *Strophariarugosoannulata* and pathogenicity tests of isolates GUCC TB1117 and GUCC TB1120 with spore suspension **a** healthy fruiting bodies of *S.rugosoannulata***b** field symptoms of green mold disease on *S.rugosoannulata***c** large stroma of the pathogen *T.strophariensis* (GUCC TB1117) **d** control, no disease after seven days of inoculation with distilled water **e–g** pathogenicity tests after spraying with 0.5 mL spore suspension (1 × 10^6^ conidia mL^–1^) **e, f** hyphal blocks and pathogen stroma (F = yellow arrow) appear on the surface of the soil after five days of inoculation **g** whole rotten fruiting bodies after seven days of inoculation **h, i** rotten fruiting bodies of *S.rugosoannulata* in the field with *T.viridistromatis* (GUCC TB1120) **j** Aggregated stroma of the pathogen *T.viridistromatis* (GUCC TB1120) with typical green symptoms **k, l** yellow arrows showing pathogen hyphal blocks and stroma appear on the surface of the soil after five days of inoculation. Scale bars: 20 mm (**a–e**); 10 mm (**f**); 20 mm (**g**); 10 mm (**h**); 20 mm (**I, j**); 10 mm (**k, l**).

Both soil inoculating groups of covering mycelial blocks and soil mixed with spore suspension of isolates GUCC TB1117 and GUCC TB1120 exhibited similar symptoms of green mold disease in the field after seven days (Fig. 1g), while the control group did not have (Fig. 1d). The green mycelia can be observed on the surface of the mushroom tray after 3–5 days and spread fast, covering the whole surface of the substrate and turning green within 10 days (Fig. 1e, f). The rate of isolates GUCC TB1117 and GUCC TB1120 infecting mushroom tray is about 50%, similar to its incidence in the field. The same fungal pathogen had been observed and re-isolated from these symptoms, which fulfills Koch’s postulates (Fig. 1).

### ﻿Phylogenetic analyses

**Figure 2.**
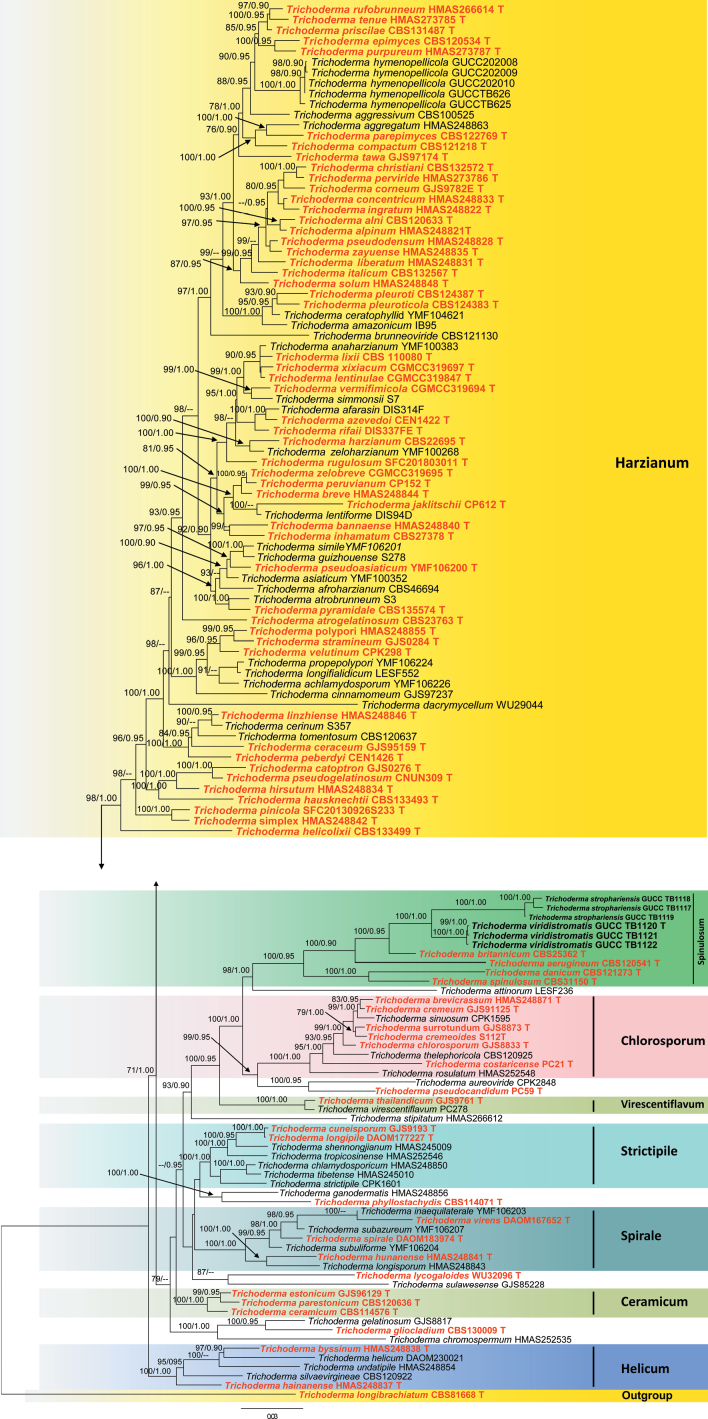
A phylogram was constructed using ML analysis, utilizing a combined ITS, *rpb2*, and *tef1-α* sequences dataset. The green-spored *T.longibrachiatum* (CBS81668) was used as the outgroup taxon following Zeng et al. (2022). The tree with the highest score according to RAxML, with a final probability value of -*InL* = 37957.575772, is displayed. Maximum Likelihood (ML) values equal to or greater than 70% and Bayesian Inference (BI) values equal to or greater than 0.90 are given above the nodes (ML values on the left side of ‘/’ in regular font and BI values on right side of ‘/’ in italics). Type strain sequences are indicated in red bold, while newly generated sequences are shown in black bold. Strain numbers for the sequences are shown in the tree following the taxon name. ‘T’ denotes ex-holotype strains.

The phylogenetic analyses were conducted using a combined dataset of nrITS, *rpb2*, and *tef1-α* sequences. A total of 128 sequences were aligned, and this resulted in a dataset consisting of 2934 nucleotides; after the ends of the individual alignments were trimmed, the size of the aligned dataset was as nrITS 610 bp, *rpb2* was 1080 bp, and *tef1-α* was 1244 bp respectively. The best-fit substitution model of each gene is ITS (TIM2+F+R4), *rpb2* (TIM3e+I+G4) and *tef1-α* (TIM+F+R4). The RAxML analysis of the combined dataset yielded a best-scoring tree with a final ML optimization likelihood value of –37957.575772. Estimated base frequencies are as follows: A = 0.233134, C = 0.285526, G = 0.253003, and T = 0.228336; substitution rates AC = 1.134637, AG = 4.477934, AT = 1.149518, CG = 1.048786, CT = 6.335323, and GT = 1.000000; proportion of invariable sites I = 0.544721; and gamma distribution shape parameter a = 0.951765. The Bayesian analysis ran 29,64000 generations before the average standard deviation for split frequencies reached 0.00998. The analysis generated 59,282 trees, from which 44,462 were sampled after burn-in, and the 99% credible set contains 35,309 trees. Our new strains belong to a distinct clade that is genetically distant from *T.britannicum*, *T.aerugineum*, *T.danicum*, and *T.spinulosum* and is divided into four subclades represented by our newly generated strains (Fig. 2). DNA base pair differences also supported the phylogenetic placements of these novel taxa (Table 2).

**Table 2.** The DNA base differences of our isolates and related taxa in different loci.

Species	Strain number	ITS (1–610 bp)	*rpb2* (611–1690 bp)	*tef1-α* (1691-2934 bp)
***Trichodermastrophariensis***	GUCC24-0002	0	0	0
***Trichodermastrophariensis***	GUCC24-0003	0	0	0
***Trichodermastrophariensis***	GUCC24-0004	0	0	0
*Trichodermabritannicum*	CBS 25362	28 (gaps: 4)	48 (gap: 0)	64 (gap: 25)
*Trichodermaaerugineum*	CBS 120541	16 (gaps: 9)	78 (gap: 0)	67 (gap: 11)
*Trichodermadanicum*	CBS 121273	25 (gaps: 8)	99 (gaps: 0)	105 (gaps: 3)
*Trichodermaspinulosum*	CBS 31150	21 (gaps: 8)	82 (gaps: 0)	108 (gap: 7)
***Trichodermaviridistromatis***	GUCC24-0005	0	0	0
***Trichodermaviridistromatis***	GUCC24-0006	0	0	0
***Trichodermaviridistromatis***	GUCC24-0007	0	0	0
*Trichodermabritannicum*	CBS 25362	17 (gaps: 5)	20 (gap: 0)	55 (gaps: 21)
*Trichodermaaerugineum*	CBS 120541	10 (gaps:10)	79 (gap: 0)	78 (gaps: 11)
*Trichodermadanicum*	CBS 121273	25 (gaps: 8)	95 (gap: 0)	113 (gap: 0)
*Trichodermaspinulosum*	CBS 31150	22 (gaps: 8)	73 (gaps: 0)	109 (gaps: 7)

### ﻿Taxonomy

#### ﻿ Trichoderma strophariensis

Taxon classificationFungiHypocrealesHypocreaceae

E. Tarafder & F.H. Tian sp. nov.

22D20AFB-BC41-5495-B40A-EB53EFFBE5CB

Fungal Names: FN 902311

Figs 3 , 4

##### Diagnosis.

*Trichodermastrophariensis* differs from *T.britannicum* by smaller stromata (0.9–2.2 × 0.8–2 mm) with dark green surface, margin free; surface finely rugose or tubercular, brownish between black ascomata; ostiolar dots absent, inconspicuous or convex to distinctly papillate measuring (27–)35–64(–90) mm diam. Additionally, it is easily distinguished from *T.viridistromatis* by its relatively larger ascospores (8.4–16.9 × 5.5–8.1 µm) and conidia (8.5–25.5 × 5.7–17.9 μm). Phylogenetically, *T.strophariensis* forms a distinct clade and is closely related to *T.viridistromatis*, *T.britannicum*, and *T.aerugineum* with 100% ML and 0.90 BYPP statistical support (Fig. 1).

**Figure 3.**
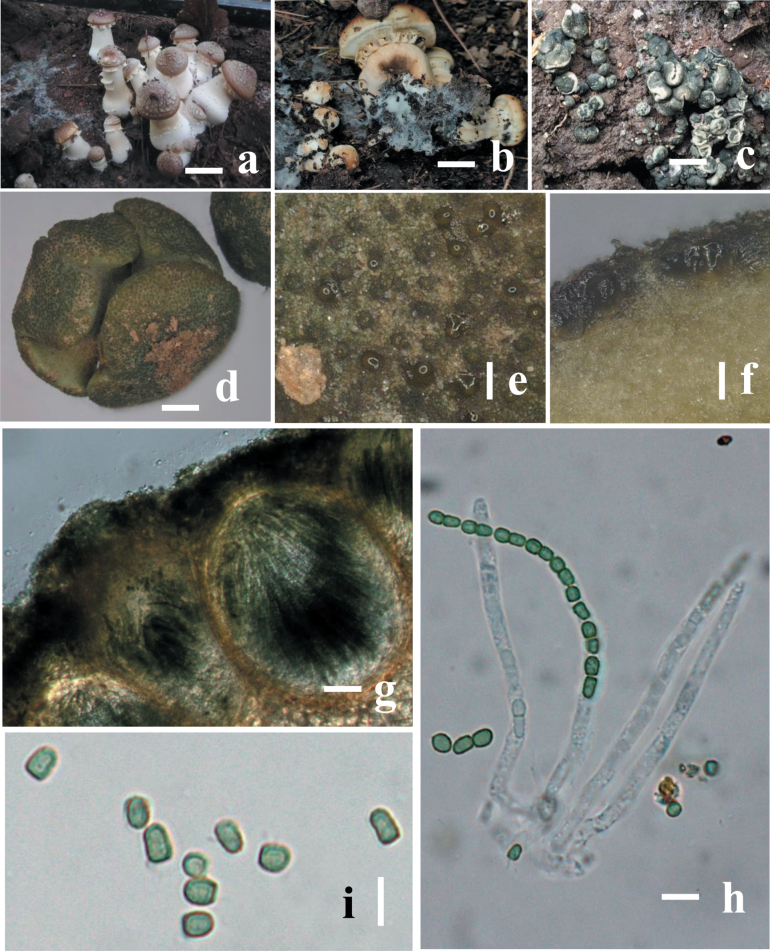
Morphology of *Trichodermastrophariensis* (HGUP 24-0001, GUCC 24-0002) **a, b** disease in the field habitat **c** fresh stromata on natural habitat **d** dry stromata **e** ostiolar dots on stromata surface **f** cortical and subcortical tissues in section **g** ascomatal tissue in section **h** asci with ascospores **i** ascospores. Scale bars: 10 mm (**a, b**); 20 mm (**c**); 100 mm (**d–f**); 50 μm (**g**); 20 μm (**h, i**).

**Figure 4.**
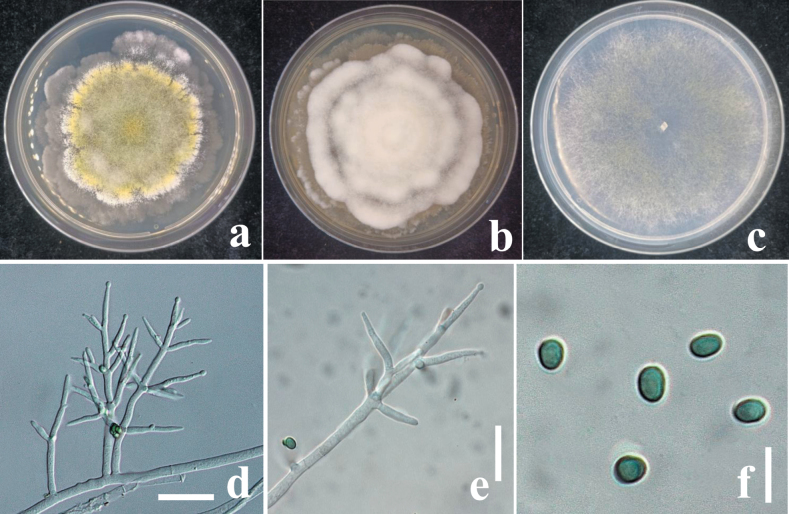
**a** cultures on MEA (five days) **b** cultures on PDA (five days) **c** cultures on SNA (4 days) **d** conidiophores **e** phialides **f** conidia. Scale bars: 10 μm (**d, e**); 5 μm (**f**).

##### Holotype.

HGUP 24-0001.

##### Etymology.

The specific epithet ‘*strophariensis*’ refers to the occurrence of the new taxon in cultivated mushrooms *Strophariarugosoannulata*.

##### Description.

***Stromata***, when fresh 1–14 mm in diameter, 1–11 mm thick (n = 10), solitary to sometimes aggregated, discoid or undulate, with brownish margin and pale red, depressed center when young, becoming reddish with rugose surface when mature. Attached to the host by hyphae, easily detached; sides often attenuated downward, surrounded at the base by white cottony mycelium when young. Surface finely rugose, tubercular, brownish between black ascomata; Ostiolar dots are convex to umbilicate, greenish, overall colors light green, darker green when dry, surface and spores green when mature. ***Ostiole*** 14–21 μm wide at apex, 41–59 μm high (n = 30). ***Ascomata*** (139–)175–295(–347) × (113–)151–248(–290) μm (n = 20), flask-shaped or sub-globose, crowded. ***Peridium*** 18–28 μm thick at the base and sides (n = 40), light brown. ***Asci*** (67–)110–146(–207) × (3.7–)5.8–7.7(–9.4) μm, stipe (3–)7–11(–18) μm long (n = 50), containing 16-ascospores, apex slightly thickened, hyaline, cylindrical. ***Ascospores*** (8.4–)9.2–11.6(–16.9) × (5.5–)6.6–7.8(–8.1) μm, l/w (1.2–)1.4–1.6(–2.1) (n = 90), green, verruculose; sub-globose, oblong, elongated, thick-walled.

##### Culture characteristics.

Optimal growth at 25 °C on all media, poor and limited growth at 30 °C, no growth at 35 °C.

On MEA and PDA growth is slow, colony creamy white, finely farinose by scant effuse conidiation; on PDA reverse brownish, surface turning greenish-brown. On MEA at 25 °C after five days colony radius 5–7 mm; colony circular, dense, thick, first whitish, becoming zonate after a few weeks, turning olive-green to brown with yellow greenish, farinose center; conidiation effuse, on short odd verticillium like conidiophores. On SNA colony radius at 25 °C after 2 weeks 6–9 mm; colony dense, hyaline, turning greenish or olivaceous from conidia. Conidiation following growth, effuse, on aerial hyphae and short odd verticillium-like conidiophores, spreading from the plug. ***Conidiophores*** simple, 1–4 level are branched and tapered at the tips, bearing few asymmetric side branches, terminated by solitary phialides of 2–3 divergent phialides. ***Phialides*** (10.5–)37–44(–55) × (1.5–)2.5–11(–12.5) μm (n = 50), mostly gregarious, cylindrical, less commonly subfusiform, often thickest near the base. ***Conidia*** (8.5–)12.5–16.4(–25.5) × (5.7–)6.5–10.7(–17.9) μm (n = 70), one-celled, variable shape and size, typically oblong and pale olive green when fully mature, sub-globose, oval or ellipsoid and hyaline when immature, straight or slightly curved, sides sometimes pinched, smooth; base often truncate, thick-walled.

##### Habitat.

On mushroom cultivated field, associated with *Strophariarugosoannulata*.

##### Distribution.

China, Guizhou Province, Guiyang City, and Liupanshui City; Guizhou City in Anshun Province.

##### Material examined.

China • Guizhou, Liupanshui City, Shuicheng District, 23°55'39.36"N, 120°11'30.64"E, on soil surfaces of *Strophariarugosoannulata* cultivated field, 16-November-2023, E. Tarafder and F.H. Tian (HGUP 24-0001, holotype); ex-type living cultures GUCC TB1117, GUCC TB1118 and GUCC TB1119.

##### GenBank accession numbers.

GUCC TB1117 (ITS: PP920011; *rpb2*: PP954941; *tef1-α*: PP954947); GUCC TB1118 (ITS: PP920012; *rpb2*: PP954942; *tef1-α*: PP954948); GUCC TB1119 (ITS: PP920013; *rpb2*: PP954943; *tef1-α*: PP954949).

##### Notes.

Morphologically, our new isolates are most similar to *T.danicum* in the size of stromata (5–20 mm) but can be distinguished by its generally smaller ascospores and conidia (Table 3); the presence of deeper color of stromata and ascospores, less pigment on media, and faster growth rate on PDA and SNA. However, our new isolates differ from *T.britannicum* by smaller stromata (0.9–2.2 mm) with dark green surfaces (Jaklitsch, 2009). In addition, it differs from other new species (*T.viridistromatis*) in producing cylindrical, less commonly subfusiform phialides (10.5–55 × 1.5–12.5 μm) and larger conidia (8.5–25.5 × 5.7–17.9 μm), typically oblong, subglobose, oval, sometimes ellipsoid and pale olive green after maturity. Phylogenetically, our isolate (HGUP 24-0001) forms an independent clade and clustering with *Trichodermabritannicum*, *T.aerugineum*, *T.danicum*, *T.viridistromatis*, and *T.spinulosum* within the Spinulosum lineage with 100% ML and 1.00 BYPP statistical support (Fig. 2). It exhibits 4% sequence differences (28/610 nucleotides, four gaps) in the ITS region, 4% differences (48/1080 nucleotides, no gaps) in the *rpb2* gene, and 5% differences (64/1244 nucleotides, twenty-five gaps) in *tef1-α* gene when compared with *T.britannicum*. Additionally, the differences between our isolate with *T.viridistromatis* are 4% (29/610 nucleotides, four gaps) in the ITS region, 4% (46/1080 nucleotides, no gaps) differences in the *rpb2* gene, and 5% (65/1244 nucleotides, twenty-five gaps) differences in the *tef1-α* gene. In contrast, the differences in our isolate with *T.danicum* are more than 4% (25/610 nucleotides, eight gaps) in the ITS region, 9% (99/1080 nucleotides, no gaps) in *rpb2* gene, and 8% (105/1244 nucleotides, three gaps) in *tef1-α* gene (Table 2). Therefore, based on both morphological and phylogenetic distinctions, *T.strophariensis* is introduced as a new species from cultivated mushrooms.

**Table 3.** Morphological comparison of *Trichodermabritannicum*, *T.aerugineum*, *T.strophariensis*, *T.danicum*, *T.viridistromatis*, and *T.spinulosum*.

Taxon (holotype)	Ascospores	Conidia	Substratum	References
*T.britannicum*	10–16 × 4.5–6.2 μm	4.7–19.3 × 4–6.2 μm	Decaying wood of broadleaf trees	Jaklitsch et al. 2014
*T.aerugineum*	8–12 × 4–6 µm	3–5 × 2–4 µm	Decaying wood	Chaverri and Samuels (2004)
***T.strophariensis***	**8.4–16.9 × 5.5–8.1 µm**	**8.5–25.5 × 5.7–17.9 μm**	**mushroom species (*Stropharia*)**	**This study**
*T.danicum*	3–5 × 2.5–4.4 µm	3–3.5 × 2.7–3 µm	On pine wood	Jaklitsch 2009
*T.viridistromatis*	3.4–5.6 × 2.4–3.3 µm	2.8–4 × 1.7–3.2 µm	mushroom species (*Stropharia*)	This study
*T.spinulosum*	5–7 × 3–4 μm	3.5–4.7 × 3–3.7 μm	On stems of *Chelidoniummajus*	Jaklitsch and Voglmayr 2015

#### ﻿ Trichoderma viridistromatis

Taxon classificationFungiHypocrealesHypocreaceae

E. Tarafder & F.H. Tian sp. nov.

6C4FE537-E9C8-585A-8384-1E1B8AF4AC09

Fungal Names: FN 902312

Figs 5 , 6

##### Diagnosis.

*Trichodermaviridistromatis* differs from *T.aerugineum* by its smaller stromata (0.5–2 mm diam, to ca. 1 mm thick in *T.aerugineum*) and bigger phialides measuring 7–23 × 2.4–4 μm in *T.aerugineum*. In addition, it is easily distinguished from *T.strophariensis* by its smaller ascospores (3.4–5.6 × 2.4–3.3 µm) and conidia (2.8–4 × 1.7–3.2 μm). Phylogenetically, *T.viridistromatis* forms a distinct clade and is closely related to *T.strophariensis*, *T.britannicum*, and *T.aerugineum* with 100% ML and 0.90 BYPP statistical support (Fig. 1).

**Figure 5.**
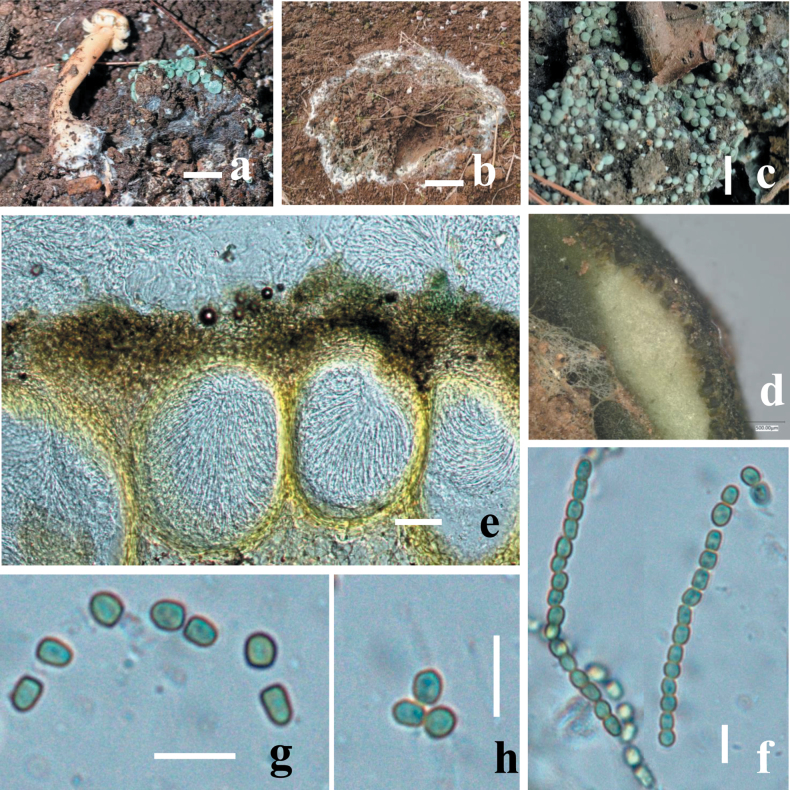
Morphology of *Trichodermaviridistromatis* (HGUP 24-0004, GUCC 24-0005) **a, b** diseased in the field, **c** fresh stromata on natural substrate **d** cortical and subcortical tissues **e** ascomatal tissue in section **f** asci with ascospores **g, h** ascospores. Scale bars: 10 mm (**a–c**); 1,000 μm (**d**); 50 μm (**e**); 20 μm (**f–h**).

**Figure 6.**
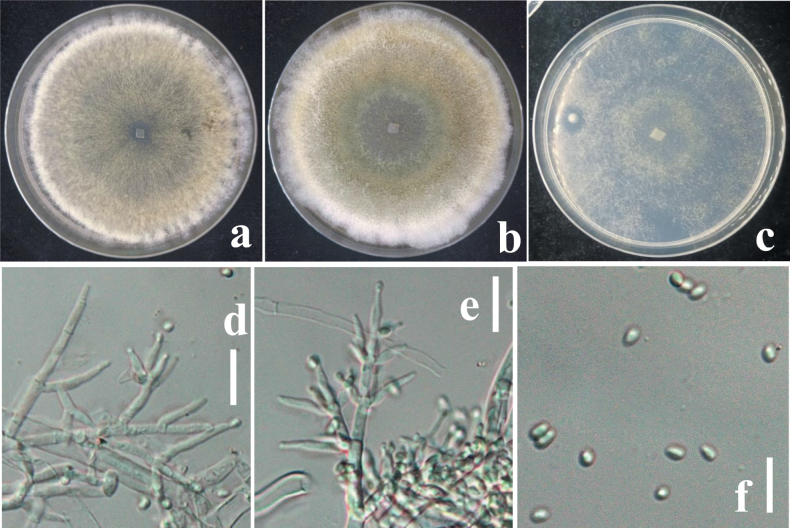
**a** cultures on MEA (five days) **b** cultures on PDA (five days) **c** cultures on SNA (four days) **d** conidiophores **e** phialides **f** conidia. Scale bars: 10 μm (**d, e**); 5 μm (**f**).

##### Holotype.

HGUP 24-0004.

##### Etymology.

The epithet “*viridistromatis*” refers to an entirely green-colored stroma.

##### Description.

***Stromata***, when fresh 1–7 mm in diam., 0.5–2 mm thick (n = 10), mostly gregarious, aggregated, discoid or undulate, becoming pulvinate, compact; outline circular to oblong; margin attached or free, surface smooth when immature without ostiolar dots, with yellowish margin and pale red, depressed center when young, becoming reddish with rugose surface when mature. Outline circular, oblong or irregularly lobed. Surface smooth, tubercular or rugose, when young finely velvety. Ostiolar dots absent, ostiolar openings sometimes visible, (16–)20–30(–32) μm (n = 30) wide, inconspicuous, pale, more distinct and shinier after rehydration. ***Ostioles*** (18–)24–30(–45) μm long, plane with the surface, (8–)12–19(–23) μm wide at the apex (n = 30). ***Ascomata*** (69–)75–85(–96) × (36–)41–55(–60) μm (n = 30), numerous, 5–7 per mm stroma length, sub-globose or flask-shaped. ***Peridium*** (7–)11–19(–22) μm (n = 60) thick at the base and sides; hyaline to pale yellowish. ***Asci*** (63–)74–81(–85) × (3.2–)4.2–5(–5.5) μm, stipe (4–)5–11(–14) μm (n = 30) long, containing 16-ascospores, apex not thickened, hyaline, cylindrical. ***Ascospores*** (3.4–)3.6–4.3(–5.6) × (2.4–)2.8–3.1(–3.3) μm, l/w 1–1.1(–1.2) (n = 34), hyaline, verruculose, single-celled, non-septate, sub-globose, oblong or slightly tapered downwards, thick-walled.

##### Culture characteristics.

Optimal growth at 25 °C on all media, poor and limited growth at 30 °C, no growth at 35 °C. Although MEA exhibited good growth, precultures were made on it.

On MEA and PDA, growth is slow, colony is creamy white, finely farinose by scant effuse conidiation; on PDA, reverse brownish, surface turning greenish-brown. On MEA at 25 °C after five days colony radius 5–7 mm; colony circular, dense, thick, first whitish, becoming zonate after a few weeks, turning olive-green to brown with yellow-greenish, farinose center; conidiation effuse, on short odd verticillium like conidiophores. On SNA colony radius at 25 °C after 2 weeks 6–9 mm; colony dense, hyaline, turning greenish or olivaceous from conidia. Conidiation following growth, effuse, on aerial hyphae and short odd verticillium-like conidiophores, spreading from the plug. ***Conidiophores*** simple, 1–4 level, are branched and tapered at the tips, bearing few asymmetric side branches, terminated by solitary phialides of 2–3 divergent phialides. ***Phialides*** (5.5–)7–10(–14) × (1.6–)2.5–2.9(–3.5) μm (n = 32), mostly gregarious, lageniform, less commonly subfusiform, not thickest near the base. ***Conidia*** (2.8–)3.1–3.7(–4) × (1.7–)2.2–2.7(–3.2) μm (n = 70), variable shape and size, typically oblong and pale yellowish green when fully mature, oval, ellipsoid and hyaline when immature, straight or slightly curved, sides sometimes pinched, smooth; base often truncate.

##### Habitat.

On mushroom cultivated field, associated with *Strophariarugosoannulata*.

##### Distribution.

China, Guizhou Province, Guiyang City, and Liupanshui City; Guizhou City in Anshun Province.

##### Material examined.

China • Guizhou, Liupanshui City, Shuicheng District, 24°55'39.936"N, 121°11'30.264"E, on soil surfaces of *Strophariarugosoannulata* cultivated field, 16-November-2023, E. Tarafder and F.H. Tian (HGUP 24-0004, holotype); ex-type living cultures GUCC TB1120, GUCC TB1121 and GUCC TB1122.

##### GenBank accession numbers.

GUCC TB1120 (ITS: PP922277; *rpb2*: PP954944; *tef1-α*: PP954950); GUCC TB1121 (ITS: PP926290; *rpb2*: PP954945; *tef1-α*: PP954951); GUCC TB1122 (ITS: PP922285; *rpb2*: PP954946; *tef1-α*: PP954952)

##### Notes.

Morphologically, our newly described taxon *Trichodermaviridistromatis* shares common characteristics with *T.aerugineum* (CBS120541) and *T.britannicum*, a species previously isolated from dead stems and leaves of *Calamagrostisepigejos*. However, *T.viridistromatis* differs from *T.aerugineum* by having smaller stromata (0.5–2 mm in diameter, compared to ca. 1 mm thick in *T.aerugineum*) and larger phialides (7–23 × 2.4–4 μm in *T.aerugineum*) and ascospores (8–12 × 4–6 µm; Table 4) (Chaverri and Samuels 2004). Additionally, it can be distinguished from *T.strophariensis* by its larger stromata (1–14 mm in diameter, 1–11 mm thick in *T.strophariensis*) and significantly larger subglobose to elongated ascospores (8.4–16.9 × 5.5–8.1 µm). In comparison, *T.britannicum* has discoid, convex to turbinate stromata surrounded by light brown radial mycelium and much larger one-celled ascospores (10–16 × 4.5–6.2 μm; Table 4) (Jaklitsch et al. 2014). The phylogenetic positions of the new taxon (Fig. 2) demonstrated that *Trichodermaviridistromatis* is closely related to *T.strophariensis*, *T.britannicum*, and *T.aerugineum*, with strong statistical support (Fig. 2). However, our isolate differs from *T.britannicum* with 3% (17/610 nucleotides, with five gaps) in ITS region, 2% (20/1080 nucleotides, no gaps) in *rpb2* gene, and 4% (55/1244 nucleotides, twenty-one gaps) in *tef1-α* gene. Moreover, the difference in our collections with *T.aerugineum* is more than 2% (10/610 nucleotides, ten gaps) in the ITS region, 7% (79/1080 nucleotides, no gaps) in the *rpb2* gene, and 6% (78/1244 nucleotides, eleven gaps) in *tef1-α* gene (Table 2). Additionally, the differences between our isolate with *T.strophariensis* are 4% (29/610 nucleotides, four gaps) in ITS region, 4% (46/1080 nucleotides, no gaps) differences in *rpb2* gene, and 5% (65/1244 nucleotides, twenty-five gaps) differences in *tef1-α* gene also supported *T.viridistromatis* to be a distinct species compared to *T.strophariensis* and *T.britannicum* respectively.

**Table 4.** Morphological comparison of *Trichodermabritannicum*, *T.aerugineum*, *T.viridistromatis*, *T.spinulosum*, and *T.strophariensis*.

Taxon (holotype)	Ascospores	Conidia	Substratum	References
*T.britannicum*	10–16 × 4.5–6.2 μm	4.7–19.3 × 4–6.2 μm	Decaying wood of broadleaf trees	Jaklitsch et al. 2014
*T.aerugineum*	8–12 × 4–6 µm	3–5 × 2–4 µm	Decaying wood	Chaverri and Samuels (2004)
***T.viridistromatis***	**3.4–5.6 × 2.4–3.3 µm**	**2.8–4 × 1.7–3.2 μm**	**mushroom species (*Stropharia*)**	**This study**
*T.spinulosum*	5–7 × 3–4 µm	3.5–4.7 × 3–3.7 µm	On stems of *Chelidoniummajus*	Jaklitsch and Voglmayr 2015
*T.strophariensis*	8.4–16.9 × 5.5–8.1 µm	8.5–25.5 × 5.7–17.9 μm	mushroom species (*Stropharia*)	This study

## ﻿Discussion

Green mold is a prevalent disease in mushroom cultivation that disrupts the average growth of mushroom fruiting bodies or mycelium and inhibits the average growth of mushrooms (Zeng et al. 2022). The discovery of two new *Trichoderma* species causing green mold disease significantly advances our understanding of fungal pathogens in mushroom cultivation. This finding underscores the urgent need for effective disease management strategies in agriculture. The pathogenicity of HGUP 24-0001 and HGUP 24-0004 on *Strophariarugosoannulata* was confirmed in controlled field tests, where both strains caused symptoms consistent with green mold disease. The rapid development of green mycelia covering the mushroom trays fulfilled Koch’s postulates. In this study, the rapid colonization of mushroom trays by green mycelia is a clear indicator of the aggressive interaction between the pathogens and the host, a situation of intense concern, leading to significant damage to the mushroom fruiting bodies. The occurrence of mold diseases affecting *S.rugosoannulata*, highlights the significant economic losses due to fungal infections in mushroom cultivation (Huang et al. 2023). The infection rate of both isolates in the mushroom trays mirrors their incidence in the field, indicating a potentially significant agricultural impact. A detailed observation of symptoms, from initial mycelial growth to full substrate colonization, provides a comprehensive timeline of disease progression and is crucial for effective disease management in mushroom cultivation (Zhang et al. 2022).

Morphological analysis of the newly identified *Trichoderma* species revealed distinct characteristics. The typical symptoms of green mold disease were greenish mycelial growth and rotting of the fruiting bodies of the mushrooms. Moreover, molecular phylogenetic analyses of the nuclear ribosomal internal transcribed spacer (nrITS) region, the second largest subunit of RNA polymerase II (*rpb2*), the partial translation elongation factor 1-alpha (*tef1-α*) provided conclusive evidence for the delineation of the two new *Trichoderma* species, and these isolates were separated from previously identified and described species of *T.britannicum*, *T.aerugineum*, *T.danicum*, and *T.spinulosum* and properly placed within the distinct clades (Fig. 2). This molecular approach not only confirmed the novelty of the species but also highlighted the genetic diversity within the genus *Trichoderma*. This study successfully identified and described two new species of *T.strophariensis* and *T.viridistromatis* as the causal agents of green mold disease in *Strophariarugosoannulata* in Guizhou Province, China.

The discovery of these new pathogens emphasizes the need for continuous monitoring and research on fungal diseases affecting economically important mushrooms. Integrating morphological and molecular identification techniques provides a robust framework for identifying and characterizing new fungal pathogens, ultimately improving disease management practices. Future studies should continue to explore the agricultural and biotechnological potential of these and other *Trichoderma* species, contributing to a deeper understanding and sustainable management of fungal pathogens in agriculture. This new pathogen can infect the mycelia of *S.rugosoannulata* at an early stage and the entire fruit body at maturity, making it a challenging competitor in the field. However, our significant findings also reveal an unexpected diversity of *Trichoderma* in China, highlighting the need for further research and inspiring future investigations.

## Supplementary Material

XML Treatment for Trichoderma strophariensis

XML Treatment for Trichoderma viridistromatis

## ﻿Additional information

### Author ORCIDs

Entaj Tarafder https://orcid.org/0000-0002-3680-3433

Zhang Wenjun https://orcid.org/0009-0005-8310-0537

Samantha C. Karunarathna https://orcid.org/0000-0001-7080-0781

Abdallah M. Elgorban https://orcid.org/0000-0003-3664-7853

Man Huilian https://orcid.org/0009-0006-7894-1873

Wu Nan https://orcid.org/0009-0006-1192-4171

Xiangyu Zeng https://orcid.org/0000-0003-1341-1004

Wang Yong https://orcid.org/0000-0003-3831-2117

Feng-Hua Tian https://orcid.org/0000-0002-6962-9531

### Data availability

Sequence data generated for the present study have been deposited in GenBank with the accession number(s) can be found below: https://www.ncbi.nlm.nih.gov/genbank/, GUCC TB1117 (ITS: PP920011; rpb2: PP954941; tef1-α: PP954947); GUCC TB1118 (ITS: PP920012; rpb2: PP954942; tef1-α: PP954948); GUCC TB1119 (ITS: PP920013; rpb2: PP954943; tef1-α: PP954949); GUCC TB1120 (ITS: PP922277; rpb2: PP954944; tef1-α: PP954950); GUCC TB1121 (ITS: PP926290; rpb2: PP954945; tef1-α: PP954951); GUCC TB1122 (ITS: PP922285; rpb2: PP954946; tef1-α: PP954952). All of the data that support the findings of this study are available in the main text.
